# Protecting Adolescents in Low- And Middle-Income Countries from Interpersonal Violence (PRO YOUTH TRIAL): Study Protocol for a Cluster Randomized Controlled Trial of the Strengthening Families Programme 10-14 (“*Familias Fuertes*”) in Panama

**DOI:** 10.1186/s13063-018-2698-0

**Published:** 2018-06-15

**Authors:** Anilena Mejia, Richard Emsley, Eleonora Fichera, Wadih Maalouf, Jeremy Segrott, Rachel Calam

**Affiliations:** 1 Instituto de Investigaciones Científicas y Servicios de Alta Tecnología, City of Knowledge, Building 219, Clayton, 0843-01103 Panama Republic of Panama; 2 The University of Manchester, Manchester, UK; 3 Kings College London, London, UK; 4 The University of Bath, Bath, UK; 5 United Nations Office on Drugs and Crime, Vienna, Austria; 6 Centre for Trials Research, DECIPHer Centre, Cardiff University, Cardiff, UK

**Keywords:** Interpersonal violence, Adolescence, Family skills training programmes, Strengthening Families Programme 10–14 (SFP 10–14), Panama, Low- and middle-income countries, Prevention

## Abstract

**Background:**

Interpersonal violence can significantly reduce adolescents’ opportunities for becoming happy and healthy adults. Central America is the most violent region in the world and it is estimated that adolescents are involved in 82% of all homicides in this region. Family skills training programmes have been designed to prevent interpersonal violence in adolescents. Several studies in high-income countries suggest they are effective. However, there are no published trials assessing effectiveness of these programmes in low- and middle-income countries (LMIC). The aim of this study is to test the effectiveness of the Strengthening Families Programme 10–14 (SFP 10–14 or “*Familias Fuertes*”) in Panama, a LMIC in Central America. An embedded process evaluation will examine the extent to which the intervention is delivered as intended, variation across trial sites, influences on implementation and intervention-context interactions. Cost-effectiveness will also be assessed.

**Methods:**

This is a cluster randomised controlled trial. The 28 townships with the highest homicide rates in Panama will be randomly allocated to implementation of SFP 10–14 alongside services-as-usual or to services-as-usual only. Approximately 30 families will be recruited in each township, a total sample of 840 families. Families will be assessed at baseline, approximately eight weeks after baseline (i.e. post intervention), six months and 12 months after. The primary outcome measure will be the parent reported externalising subscale of the Child Behaviour Checklist at T3 (i.e., which is approximately 12 months after baseline). For the process evaluation, recruitment, attendance, fidelity and receipt will be measured. Qualitative interviews with facilitators, trainers, parents and adolescents will explore barriers/facilitators to implementation and intervention receipt. For the cost-effectiveness analysis, service use information will be gathered from parents and adolescents with a three-month recall period. Costs and consequences associated with implementation of the intervention will be identified.

**Discussion:**

This trial will be the first to evaluate SFP 10–14 in a LMIC. Results have the potential to guide public policies for the prevention of interpersonal violence in Central America and beyond.

**Trial registration:**

ISRCTN Registry, 14023111. Registered on 13 July 2017.

**Electronic supplementary material:**

The online version of this article (10.1186/s13063-018-2698-0) contains supplementary material, which is available to authorized users.

## Background

Central America is the most violent region in the world in terms of interpersonal violence (i.e. child maltreatment, intimate partner violence, youth gang violence and crime) [1]. The homicide rate due to interpersonal violence is 28.5 per 100,000 inhabitants in comparison with 10.9 in Africa, the second highest region [2]. Young people in Central America are disproportionally affected. According to the 2014 report ‘Health for the World’s Adolescents’, interpersonal violence is the leading cause of adolescent mortality and morbidity in Central America [3]. Those aged < 29 years in upper-middle income countries, such as Panama and Costa Rica, are involved in 82% of all homicides [3].

Perpetration and being a victim of interpersonal violence early in life is not only associated with death and physical injuries, but also with behavioural, mental and social consequences which create a burden for health and justice systems [4]. For example, interpersonal violence is associated with risky sexual behaviours, poor school performance, alcohol and drug abuse, which in turn are risk factors for health difficulties such as early pregnancy, HIV, cancer and cardiovascular diseases later in life. Short- and long-term health consequences of interpersonal violence harm individuals, families and communities, compromise economic development of countries in Central America, and place a great burden on international aid from high-income countries [2]. Violence reduction is key for improving worldwide health.

### The role of the family in violence prevention

Healthy family functioning is one of the most crucial factors protecting adolescents from interpersonal violence. Recent reports from the World Health Organization [5, 6] suggest that family social support [7], family cohesion [8], parental monitoring and non-hostile parenting practices are all protective factors of interpersonal violence [9]. While pathways through which family variables lead to perpetration of interpersonal violence have not been definitively described, poor parental communication and problem-solving skills plus family stress (e.g. divorce, high inter-parental conflict) are associated with the highest levels of offences, arrests and convictions in youth [10]. Poor parenting can be understood as a stressor and, in combination with other family stressors (e.g. divorce, domestic abuse), it accentuates problem behaviours of adolescents. On the other hand, good parenting may serve as a buffer for family stressors.

Based on this literature, family skills training programmes have been developed since the 1980s and are considered among the most effective strategies to prevent interpersonal violence [11]. They are designed to strengthen family protective factors such as communication, trust, problem-solving skills and conflict resolution, and often include opportunities for parents and children to spend positive time together, as ways to strengthen the bonding and attachment between the two.

Most family skills training programmes are mainly used for universal prevention. In other words, they target whole populations (e.g. entire schools or neighbourhoods) without any specific consideration to the risk level present. The idea is that anyone can benefit from prevention efforts with a health promotion orientation and the approach benefits from being non-stigmatising.

### The Strengthening Families Programme 10–14

The Strengthening Families Programme 10–14 (SFP 10–14) is one family intervention with evidence of effectiveness for reducing youth violence in the United States [12]. SFP 10–14 is skill-oriented with underpinnings in theories of bio-psychosocial vulnerability [13] and resilience [14]. It was developed to address risk and protective factors at the individual and family level. It is offered as a seven-session universal package (i.e. targeting all levels of risk) for the transition from childhood into early adolescence (ages of 10–14 years).

Blueprints on Violence Prevention ranks SFP 10–14 as a preventive package with ‘evidence of benefits-minus-costs’ and ‘promising’ impact because of its clear logic model, the validity and reliability of its evaluation findings, its significant positive effects on intended outcomes and its readiness for dissemination [15]. According to its logic model, developing skills in adolescents and parents leads to short-term family and individual changes such as better family functioning, less parental stress, better skills for social interaction in youth and less favourable attitudes towards violence and substance use. These proximal outcomes could then lead to long-term public health changes such as reduced criminality, delinquency and less substance use in communities.

Evaluations of SFP 10–14 in the United States suggest medium to high effect sizes of the programme on adolescent exposure to illicit substance use and young adult lifetime substance use (d = 0.40–0.50). However, there is only one trial evaluating effects of the programme on aggressive and hostile behaviours of adolescents. This trial suggests significant improvements in observer ratings of adolescent aggressive and hostile behaviours when interacting with their parents, in family-member reports of aggressive and hostile behaviours, and in adolescent self-report of aggressive and destructive conduct across settings at 1.5, 2.5 and 4 years after the programme [12].

Besides studies in the United States, SFP 10–14 has been evaluated in Germany [16], Wales [17], Poland [18] and Sweden [19], but until now no evaluation has been conducted in a low- and middle-income country (LMIC) where interpersonal violence rates are high. In addition, more trials of SFP 10–14 are needed, given that no evaluations in high-income countries other than the United States have found positive effects of the programme on alcohol use-related outcomes or on family relationships and functioning.

### The Strengthening Families Programme 10–14 in Panama

Since 2009, the United Nations Office on Drugs and Crime (UNODC) has invested significantly in promoting evidence-based prevention in LMICs using a top-down and a bottom-up approach [20]. Their top-down approach involves engaging directly with policy makers in order to change their views and priorities and ensure their understanding of prevention principles guided by the International Standards on Drug Use Prevention. This entails: (1) explaining the aetiology upon which prevention interventions should be based; (2) explaining the science of prevention; (3) identifying effective evidence-based prevention interventions and the characteristics that make them effective; (4) identifying ineffective interventions; and (5) indicating what makes an effective system of prevention interventions. The aim of UNODC’s approach with policy makers is to ensure service providers at the ‘bottom’ level have access to evidence-based interventions. On the other hand, UNODC’s bottom-up approach focuses on piloting evidence-based preventive interventions adapted to national needs and documenting evaluation reports on their process of implementation, effectiveness and cost-effectiveness [20]. The main social institution of this bottom-up approach is the family. Among the family skills programmes being piloted in Central America is SFP 10–14. Panama was the first country from the Central American region where SFP 10–14 pilots were initiated with UNODC’s support.

The SFP 10–14 programme was originally translated and adapted to the Latin American context by the Pan American Health Organization (PAHO) in close collaboration with its developers. The culturally adapted version of SFP 10–14 was referred to as *Familias Fuertes*. However, for its pilot in Panama, UNODC undertook a cultural review of *Familias Fuertes* and conducted further adaptations to ensure its fit to the local context. These adaptations consisted of changing only names and examples. There were no changes affecting the structure, content or the order of the sessions. Since 2009, the intervention has been delivered to 432 Panamanian families and there are approximately 152 accredited facilitators and 27 local trainers.

UNODC conducted pre-post evaluations of SFP 10–14 in Panama, Honduras and Guatemala that suggested reductions in parental violence towards adolescents and improvements in adolescents’ attitudes towards others after participation in the programme. In addition, in 2012 qualitative evaluations with 30 Panamanian parents who took part in the intervention were conducted to explore acceptability and satisfaction. Results were positive, suggesting the intervention was satisfactory to parents and addressed their concerns in a culturally sensitive manner [21, 22].

Given the lack of rigorous data regarding the effectiveness of SFP 10–14 to prevent violence in LMIC and building on previous efforts by UNODC to adapt and implement the programme in Central America, the main aim of the present project will be to test effectiveness of the culturally adapted version of SFP 10–14 in Panama. We will build on UNODC’s previous investments by evaluating implementation of SFP 10–14 in existing health and educational services across Panama in close partnership with local institutions. This will be the first implementation trial of SFP 10–14 in a LMIC. We chose Panama for this evaluation because, first, UNODC has its main physical base for Central America and the Caribbean in this country thus easing communication/impact across the Region. Second, Panama is an ideal country for implementation of the programme given its strong governmental support, specifically from the Ministry of Health and Ministry of Education who agreed to commit staff time and infrastructure for this trial.

In sum, the aims of the study are: (1) to test the effectiveness of SFP 10–14 in reducing youth aggressive and hostile behaviour, as reported by parents and adolescents, when implemented via health and educational sites in Panama; (2) to assess the implementation process of SFP 10–14, specifically, implementation fidelity and how these processes vary across sites in order to optimise its scaling up and sustainability should the intervention be shown to be effective; and (3) to assess the cost-effectiveness of SFP 10–14 in Panama.

## Methods/design

**Fig. 1:**
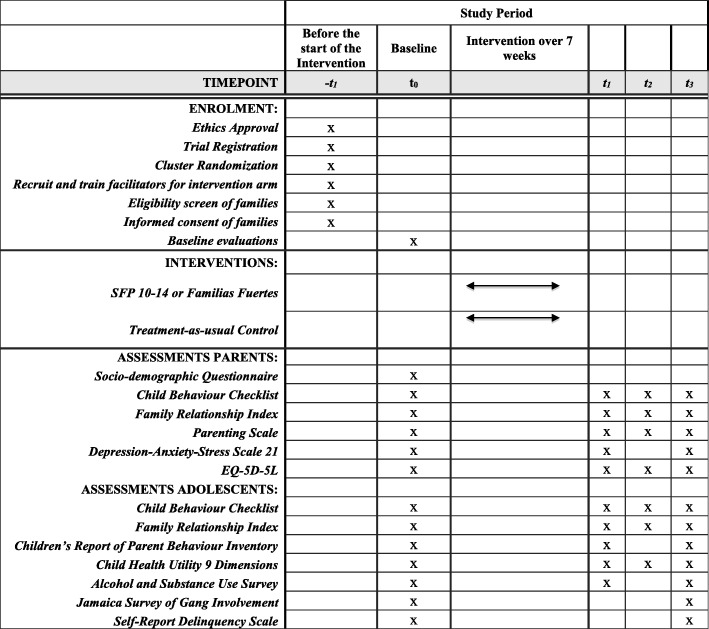
Spirit figure

This will be a cluster randomised controlled trial (RCT) with two arms: (1) implementation of SFP 10–14 in health and educational services plus services as usual (*n* = 14 clusters); or (2) services as usual only (*n* = 14 clusters). Clusters will be state-owned clinics or schools located in the 28 townships (i.e. *corregimientos* which are political subdivisions within Districts) with the highest homicide rates in the Districts of Panama Centre, Panama East, Panama North and San Miguelito. Out of the 41 townships in these four Districts, the 28 townships with the highest number of homicides per 10,000 inhabitants will be selected for randomisation. The most recent homicide data that will be used are for the years 2015 (whole year) and 2016 (only January until August). The Office of Criminal Statistics (SIEC) at the Ministry of Security will provide homicide data. Size of townships is in the range of 3000–100,000 inhabitants, with an average 8% of the population aged 10–14 years. To reduce contamination, only one site (clinic or school) in any given township will be selected and randomised. A SPIRIT checklist is attached as an Additional file 1 to this manuscript and the SPIRIT Fig. 1 shows the study design.

### Participants

A team of approximately four staff from the Ministry of Health or Education working in selected sites will invite families from those who access their services regularly or more widely from the community to take part in the trial. These staff will be doctors, nurses, psychologists, social workers or health promotion staff in the case of clinics, and teachers in the case of schools. Key inclusion criteria for participation of families in the trial will be: (1) families with a male or female adolescent aged 10–14 years; (2) at least one primary caregiver and one child aged 10–14 years are willing to attend the programme together within a fixed time period; and (3) the ability to speak Spanish (literacy aid will be provided to parents or children who cannot read or write). Key exclusion criteria for participation of families in the trial will be: (1) families in which children and both parents live separately (e.g. the child is in care); (2) families that have participated in SFP 10–14 previously; and (3) families that have taken part in any other family skills training programme in the last 12 months.

### Clusters

Once the 28 townships with the highest rates of interpersonal violence are identified, the administrative counterpart at Ministry of Health (MINSA) will identify a clinic with specialist services for adolescents within each township that meet key inclusion criteria (defined below). These clinics will become clusters for the trial. Only clinics with specialist services for adolescents will be considered for this trial because they are currently the only ones with enough staff for delivering the intervention. In addition, these are the only services that allow consistent access to the adolescent population within townships and have well-established mechanisms for following them up. In the case that there is no clinic with specialist services in a given township, the Ministry of Education (MEDUCA) will be approached in order to identify a suitable school that meets key inclusion criteria. These schools will become clusters for the trial. Clinics and schools have universal reach in townships in Panama.

Key inclusion criteria for a clinic or school to be selected as cluster will be: (1) offering specialist health services or educational services to adolescents aged 10–14 years; (2) being located within one of the 28 townships with highest homicide rates; (3) having at least four permanent staff willing to be trained to recruit families and deliver the intervention; (4) permanent staff available to recruit and deliver the intervention are doctors, nurses, psychologists, social workers or health promotion staff in the case of clinics, and teachers in the case of schools; and (5) having physical space available to deliver the intervention. Key exclusion criteria for not selecting a clinic or school as cluster will be: (1) not offering specialist health services or educational services for adolescents; (2) not having enough staff available to recruit families and deliver the intervention; and (3) not having physical space to deliver the intervention.

### Sample size

For the main trial, the sample size takes into account the intra-cluster correlation coefficient, the maximum cluster size, the expected effect, dropout and the power of the study, and was performed using the *clsampsi* command in Stata. We do not have references to support what the effect sizes would be as this is the first cluster RCT of Familias Fuertes. The effect sizes were chosen as conservative estimates for the ICC. We assumed an intra-cluster correlation of ρ = 0.1 in each arm and a maximum of three groups (30 families) at each site. We assume 90% power for a standardised effect size of 0.5 (based on the primary outcome) with significance level 0.05. The optimum design requires 13 clusters in each arm and 780 families. The estimated dropout rate is 7%. To account for dropout of families, we will recruit an additional site in each arm (we do not anticipate any cluster-level dropout). This leads to a final sample of 28 clusters and 840 families, recruited at baseline. In practice, if a larger ICC is found, this will reduce power to detect the same effect size; an ICC of 0.2 would have 71% power for an effect size of 0.5.

### Recruitment of families

The same recruitment strategy will be used for both arms. Approximately Four selected staff in intervention and control sites will recruit families universally into the study from those who access their services regularly or more widely from the community. Recruitment will take place via referral of families that are accessing services and via open invitations in the community (e.g., in churches and municipalities). These recruiters will be teachers in the case of school sites and nurses, social workers, psychologists, health promotion staff and doctors in the case of clinics. Families in both arms will be compensated at each assessment session to increase retention (USD 4.50 per family). An average hourly wage in Panama is USD 2.47 so compensation will cover 1 h of work plus travel. Families will also receive promotional materials (e.g. keychains, magnets, pens) to increase motivation and retention.

### Randomisation

A minimisation algorithm will be used to ensure balance across arms in terms of: (1) the population size of townships; (2) baseline levels of interpersonal violence in townships; and (3) type of site (e.g. clinic or school). Given that this is a real-world implementation trial that involves training a limited number of staff embedded in selected sites, sites need to be randomised before families are recruited into the study. We are aware randomisation of clusters before recruiting participants can influence recruitment and dropout in the services-as-usual (SAU) arm. To minimise these issues, we have included costs for compensating families per assessment.

### Blinding

This is an open trial. Research assistants, staff at clusters and families will be aware of participants’ allocated condition during the trial. Those coding data will be un-blinded to group allocation, but those analysing data will be blinded.

### Intervention condition

Families in the intervention arm will receive SAU plus SFP 10–14. SFP 10–14 will be delivered in groups of approximately ten families (a minimum of six and a maximum of 16 families). The intervention will only be available in selected townships via the trial. In this trial, we will use a ‘universal’ approach in which facilitators will recruit families from the general population and not only those at risk. In other words, they can recruit families from those who access their services generally as well as more widely from the community. The programme comprises seven weekly sessions of 2 h each. Parent and adolescent sessions are conducted separately in the first hour, followed by a second hour together as a family. The first hour focuses on skills, with the second hour designed to recognise family strengths and practice skills covered in the first hour. The intervention addresses three broad areas: family functioning, including communication between parents and children; strengthening parental skills; and helping young people to develop new skills in relation to resisting peer pressure, stress management and goal setting.

MINSA/MEDUCA will select staff to be trained as facilitators of SFP 10–14, trying to identify as much as possible staff who might deliver beyond the trial (i.e. engaged and enthusiastic staff with previous experience working with families). In intervention sites, four staff per site will be trained. We will train 56 new facilitators in two training groups of 28 each. For each training group, three experienced Panamanian trainers will train new facilitators of the intervention. New facilitators will deliver the intervention to a first cohort of families. A cohort is made of one group of approximately ten families per site (140 families in total; ten in each of the 14 sites). After delivery of the intervention to the first cohort of families, the best facilitator per team (i.e. the most committed, empathic, dynamic and with the best skills to manage families) will be trained to become trainer of others. Trainers within each team will be trained to train new facilitators and thus sustain the intervention in the future. Experienced international trainers will be in charge of training trainers.

### Control condition

The comparison condition will be SAU only. There will be no defined programme of usual care in control sites, though we will measure what this arm receives. The existing services available to families and adolescents in clinics and schools will continue throughout the trial. A team of approximately four permanent staff at control sites will be selected to recruit families throughout the trial. In order to ensure that all families have access to the intervention, those in the control group will be offered the intervention at the end of the trial, following the final assessment (i.e. at 12-month follow-up) but only if the intervention is found to be effective.

### Data collection methods

There will be three assessment procedures. Parents and adolescents could decide to complete assessments using paper questionnaires in face-to-face sessions. For this purpose, research assistants will coordinate group assessment sessions (per wave of ten families) conducted at sites. Although we do not expect many illiterate parents given the local literacy rate (98%), research assistants will also be trained to conduct individual read-aloud interviews in face-to-face sessions. Assessments could also be conducted via telephone sessions if preferred by the parent or adolescent. In this case, research assistants will read aloud questionnaires over the telephone. Finally, there is no postal system in Panama, but follow-up questionnaires could also be delivered home in which case parents/adolescents will have seven days to return them to the research team in a sealed envelope or the research team could collect them from families' homes.

### Outcome measures

The Spanish version of all questionnaires will be used. The primary outcome will be Problem Behaviours as measured with the Externalising subscale of the Child Behaviour Checklist (Parent Version) for children aged 6–18 years [23] that measures rule-breaking and aggressive behaviour. The primary endpoint will be T3 that is approximately 12 months after baseline. The Externalizing subscale of the CBCL Parent version consists of 35 items responded by parents using a scale from 0 to 2 (0 = not true, 1 = somewhat or sometimes true, 2 = very true or often true). The questionnaire takes 10 min to complete.

For parent-reported secondary outcome measures, family functioning will be measured with the Family Relationship Index (FRI) [24]. The FRI is a 27-item uni-dimensional measurement of the quality of social relationships in the family environment as determined by cohesion, expressiveness and conflict. Participants respond True or False to each item. Parental Discipline will be measured with the Parenting Scale (PS) [25]. The PS is a 7-point Likert-scale 30-item questionnaire that measures parenting practices in three subscales: laxness; over-reactivity; and hostile parenting. Laxness refers to a parent’s inconsistency or permissive parenting, while over-reactivity refers to a parent’s harsh or punitive parenting. Hostile parenting refers to the extent to which a parent hits, curses or insults their child. Parental stress will be measured with the Depression-Anxiety-Stress Scale 21 (DASS-21) [26]. DASS-21 is a 21-item self-report questionnaire designed to measure the severity of a range of symptoms common to both depression and anxiety. The individual is required to indicate the presence of a symptom over the previous week. Each item is scored from 0 (did not apply to me at all over the last week) to 3 (applied to me very much or most of the time over the past week). Quality of life will be measured with the EQ-5D-5 L [27], which assesses mobility, self-care, usual activities, pain/discomfort and anxiety/depression. Each dimension has five levels: no problems; slight problems; moderate problems; severe problems; and extreme problems. The respondent is asked to indicate his/her health state by ticking in the box against the most appropriate statement in each of the five dimensions. We will use the validated Spanish version provided by EuroQoL.

In terms of adolescent-reported secondary outcome measures, problem behaviours will be measured with the Externalising Subscale of the Youth Self-Report CBCL (YSR) [23]. It is composed of 32 items that are responded on a 0–2 scale (0 = not true, 1 = somewhat or sometimes true, 2 = very true or often true). As in the parent-reported version of the CBCL, the YSR assesses rule-breaking and aggressive behaviour. Family functioning will be measured with the Family Relationship Index [24]. Parental discipline will be measured with the Children’s Report of Parent Behaviour Inventory. This instrument has 52 items to evaluate the relationship of the child with his/her mother and 52 items to evaluate relationship with his/her father. Items are responded on a 1–3 scale (1 = never, 2 = sometimes, 3 = often). Quality of life will be measured with the Child Health Utility 9 Dimensions, which is a paediatric generic preference-based measure of health-related quality of life. It allows the calculation of quality-adjusted life years (QALYs) for use in cost-utility analysis. It assesses nine dimensions with five response options each. We will use the validated Spanish version provided by Scharr at the University of Sheffield. Substance misuse will be measured with ten items from the Health Behaviour for School-Aged Children Questionnaire (HBSC). These items measure frequency of smoking cigarettes and e-cigarettes, frequency of use of different types of alcoholic drinks, age of initiation of alcohol use and smoking, marijuana intake and use of other drugs. Gang involvement will be measured with the Jamaica Survey of Gang Involvement from the Jamaica Youth Survey [28]. While the full survey is 107 items to measure five core competencies, for this study we will only use four items that measure previous gang history. Delinquency will be measured with the Self-Report Delinquency Scale [29]. This instrument has 39 items in which adolescents respond how many times in the last 12 months have they engaged in delinquent and criminal activities. They are able to choose from (a) once a month, (b) once every 2–3 weeks, (c) once a week, (d) 2–3 times a week, (e) once a day to (f) 2–3 times a day.

### Participant timeline

#### First meeting with families

Staff at clinics and schools will send home invitation letters and Participant Information Leaflets (one version for the parent and one version for the adolescent) to those families who access their services regularly and to those from the township recruited openly (e.g., from churches, municipalities) and meet inclusion criteria. Invited families will be asked to attend an informative meeting approximately three days after, in which research assistants will explain the project and what it entails. All families (control and intervention) will be given the same information at this point. First, it will be explained that if they are in an intervention township, they will need to attend seven family sessions, followed by assessments immediately after the last session (post-intervention), approximately six months and 12 months after. On the other hand, if they are in a control township, they will only complete assessments to see how they are doing throughout time and will only receive the intervention at the end of the trial (approximately 12 months later) if it is shown to be effective. Families that agree to take part will be screened and registered into the trial. Both parents and adolescents will sign an informed consent and complete baseline measures. Families in the intervention group will be given an invitation card for the first intervention session that will take place the following week. All families will agree with the facilitator on the best time/day of the week to run the intervention and assessment sessions from a range of options (e.g. evenings after work, Saturdays). Families in the control group will be given an invitation card for the post-intervention assessment approximately eight weeks after. They will also all agree on the best time/date to run these assessments.

#### Follow-ups at post-intervention, six and 12 months after

**Fig. 2:**
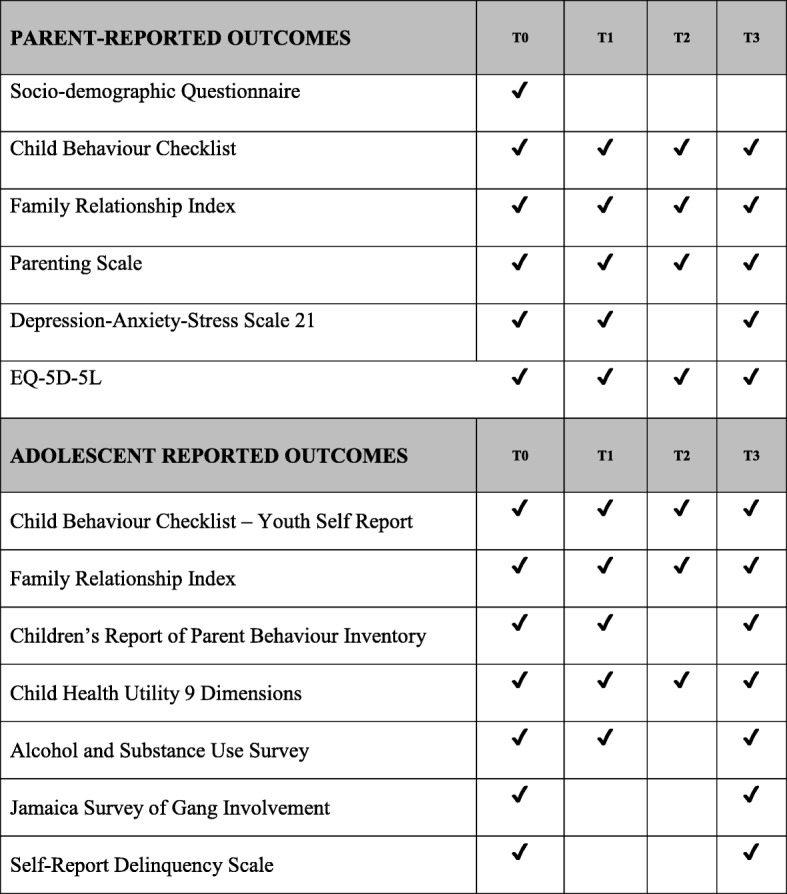
Assessment instruments per time point

Follow-up assessments will take place approximately 8–12 weeks after baseline (i.e. post intervention), 4–8 months after baseline and 10–14 months after baseline. Assessments will be conducted in face-to-face sessions (i.e. in groups alongside ten other families from their cohort), in telephone sessions facilitated by a research assistant or individually at home and returned to the research team a week after. In Fig. 2, we summarise the outcome measures that will be used at each assessment point.

### Statistical methods

We will follow CONSORT guidelines for reporting and analysis of cluster RCTs [30, 31]. Participant flow will be reported and analyses will be conducted on the intention-to-treat (ITT) population; all participants randomised will be included regardless of non-compliance with protocol or withdrawal from the study. Analyses will postdate final follow-up assessments, with due consideration of potential biases from loss to follow-up. We will use linear mixed effects models with random intercepts for site and participants will be fitted to the repeated measures to estimate treatment effects for the primary and secondary outcomes. Covariates will include the corresponding baseline outcome measure and minimisation factors. We will allow for missing outcome data under the Missing At Random (MAR) assumption and check the sensitivity of treatment effect estimates to departures from MAR. We will conduct a dose response analysis to estimate effects for number of sessions attended using instrumental variable methods.

### Process evaluation

The aims of the process evaluation are threefold: (1) to assess the extent to which SFP 10–14 is delivered as intended and describe variation across trial sites and over time; (2) to identify key influences on implementation and the role played by intervention–context interactions; and (3) to determine the sustainability of the intervention beyond the trial-funded period and what systems and structures might be needed for longer-term implementation.

#### Implementation fidelity

Following the framework proposed by Linnan and Steckler [32], the process evaluation will assess: (1) intended and actual intervention and trial recruitment rates; (2) dose delivered, defined as the number of intended programmes (and their constituent sessions) which take place; (3) fidelity, which will encompass coverage of intended programme content by facilitators, implementation quality, adherence to staffing requirements (numbers, consistency), and group size and composition; (4) dose received (engagement by families); (5) programme reach – the number of sessions which trial arm families attend; and (6) provision and quality of intended inputs (suitability of programme venues, arrangements for family transport, refreshments, etc.). Data on recruitment, dose delivered, reach, staffing and group size/composition will be collected by trainers/facilitators as part of routine monitoring and will be made available to the process evaluation. Trainers/facilitators will self-assess fidelity of all sessions using tools produced by the programme developers and used in previous RCTs of SFP 10–14 [12, 33]. They will also record information on engagement by families, provision of inputs for each session and note any problems/challenges encountered during implementation. A research assistant will observe two sessions in each of fourteen intervention trial sites per cohort of recruitment (3 cohorts). The researcher will measure fidelity by using the same scoring systems as trainers/facilitators in order to conduct reliability checks. They will also collect qualitative data on group dynamics and management. Qualitative interviews with trainers (*n* = 14), facilitators (*n* = 28) and directors/policy makers (*n* = 13) will explore implementation context, including the provision of other services within local settings. Interviews with parents/carers (*n* = 15) and adolescents (*n* = 15) in the trial arm will also explore receipt of the intervention and its perceived value and acceptability to them.

#### Key influences on implementation

Qualitative interviews with trainers/facilitators/directors/policy-makers will allow us to investigate the factors which influence implementation of SFP 10–14 (*Familias Fuertes*), particularly how the interaction between the intervention and local delivery systems may explain variations in fidelity, recruitment, etc. across trial sites and over time. We will use May’s [34] Extended Normalisation Process Theory (ENPT) [34] as a framework to understand the role of practitioner agency, organisational readiness and social systems/structures in shaping implementation processes (both in terms of barriers and facilitators), and to explain key patterns in the quantitative findings on fidelity and other aspects of delivery. In line with ENPT we will examine: (1) practitioner agency, and the extent to which individual programme staff and other key actors within delivery systems value, and are committed to implementing SFP 10–14 as intended; (2) the feasibility of implementing the intervention (e.g. the workability of facilitator roles and programme activities) and whether it can be integrated within existing delivery systems; and (3) the capacity within social systems to provide the financial resources, inter-agency coordination, and favourable norms and expectations necessary for implementation to take place.

#### Intervention sustainability

Interviews with trainers/facilitators/directors/policy-makers will examine the extent to which SFP 10–14 has become embedded within local delivery systems, the levels of support it enjoys from individual practitioners and partner agencies/potential funders, and the feasibility of delivering the intervention as intended beyond the end of the trial. Through integrating quantitative data on implementation fidelity and qualitative findings on processes shaping delivery, we will identify the key conditions necessary for the programme to be delivered as intended (e.g. material resources, support from partner agencies), and the systems and structures which may be needed for implementation in Panama beyond the funded trial period. Where barriers to implementation and the embedding of SFP 10–14 within delivery systems are identified we will examine whether and how these might be overcome. We will present emerging findings to programme trainers, senior managers from township/district agencies, and national government policy makers, to refine our understanding of organisational readiness and strategic support for continued implementation of SFP 10–14 in Panama.

### Economic evaluation

The aim of the economic evaluation is to assess the value for money offered by the program. To do so, we will consider the payer and societal perspectives, encompassing health and social services, education and criminal justice, and families participating in the programme.

#### Costs

It may not be possible to measure all of the costs and benefits associated with SFP 10–14, but we aim to provide a full identification of the most important ones. Costs will be determined in three areas: (1) variable and fixed costs of setting up, organising and operating the programme (e.g. materials, staff wages); (2) resources utilised by adolescents and families to attend (e.g. out-of pocket expenses); and (3) cost to other government services (including those due to interpersonal violence, drug use, healthcare services and education). Direct variable and fixed costs (area 1) will be recorded at the start of the programme. A weekly cost diary and questionnaire will be developed locally and will be completed by facilitators to keep track of operating costs (area 2). These should record actual session time, home visits/telephone calls, travel costs, space rentals (if any), stationery, equipment (e.g. computers) and travel costs.

The Client Service Receipt Inventory (CSRI) [35] will be adapted for Panama in order to gather information on service use (area 3). The CSRI is a resource utilisation collection tool used in the evaluation of other early childhood interventions [36]. While the central tenets behind the construction of the CSRI do not vary regardless of where an economic evaluation is undertaken, it is important to make sure that the CSRI is appropriate for Panama. There are two challenges to amending the CSRI for Panama. First, service systems are very different in Panama from other countries where the SFP 10–14 has been implemented such as the U.S. These different services may be provided by different agencies or draw from different funding streams. Second, service titles might also differ from other contexts. Our strategy in adapting the CSRI to Panama will involve a literature search as well as consultation of local parties. First, we will draw on existing international versions published by the Personal Social Services Research Unit (University of Kent, 2017). Second, we will consult the Database of Instruments for Resource Use Measurement (DIRUM) for relevant instruments by categories of age and intervention. After drafting the English version of the CSRI, we will ask local researchers to translate it into Spanish. Finally, we will submit the draft version of the CSRI to a focus group composed of (non-participating) families and adolescents, school directors, police forces, social services and test its feasibility, relevance, completeness and clarity. We will use a recall period of three months that is deemed sufficient to obtain a representative picture of service use, while also being sufficiently recent to allow accurate responses on frequency and nature of contacts. Unit costs for healthcare services will be obtained from WHO-CHOICE unit costs estimates for Panama and from our local partners, MINSA. Unit costs for other government services at the township level (such as those related to crime and education) will be obtained from Ministry of Justice and MEDUCA.

#### Economic evaluation methods

The within trial economic analysis has two components. First, a cost-effectiveness analysis of the intervention controlling for potential confounders will be performed whereby incremental cost-effectiveness ratios will be estimated relative to usual care. This analysis will take the healthcare payer perspective. The confidence interval will be generated using bootstrapping with 1000 replications. Costs will be differentiated between research and programme-specific components so as to attribute them correctly to the intervention programme. For instance, costs incurred by agencies will be clearly identified as they might benefit from resources utilisation as well as to allow inter-sectoral comparisons. The primary outcome for the cost-effectiveness analysis will be the EQ-5D-5 L [27] and a secondary cost-effectiveness analysis will be performed using the Child Health Utility 9 Dimensions [37]. The Spanish versions of both instruments will be used and QALYs calculated from individuals’ responses using the area under the curve method. As the SFP 10–14 has the potential to impact upon outcomes beyond health, we will also perform a secondary analysis of the costs and benefits of the intervention on non-health outcomes, such as crime and education, from a societal perspective. We will estimate the internal rate of return (IRR) to evaluate the desirability of investments in the SFP 10–14. The IRR allows us to determine the rate at which an investment breaks even. This approach has been taken in the evaluation of other childhood interventions such as the well-known Perry Pre-School programme in the United States [38].

A battery of sensitivity tests will follow the economic analyses. A probabilistic sensitivity analysis [35] will assess likelihood that the intervention would be considered cost-effective at a range of different willingness to pay thresholds. Key thresholds include the WHO recommendation of 1–3 times GDP per capita and a threshold range of USD 5352–12,083 adjusted by Purchasing Power Parity previously estimated for Panama [39]. Because there are advantages and disadvantages for decision-makers to using these thresholds, various cost-effectiveness thresholds should be incorporated in studies conducted in LMICs [39]. A range of one-way sensitivity analyses will be conducted which will vary cost (e.g. excluding non-recurrent costs) and effectiveness inputs and examine sub-groups. The results will inform further modelling of the long-term cost-effectiveness of the intervention beyond the trial period. We will estimate the IRR under a series of different assumptions: (1) including health outcomes; (2) varying the estimated social costs of crime; and (3) in the event that the only benefit of the programme is crime reduction. We will determine at which rate the investment would break even under these assumptions.

### Data management

Digital data will be entered into a database that will be managed securely in Panama and the UK throughout the project. Anonymised and sensitive data will be stored in Panama on laptops and at the University of Manchester Research Data Management Service (RDMS) via secure, encrypted transfer using the University of Manchester’s ZendTo service. The RDMS provides robust, managed, secure, replicated storage and allows researchers to store, manage and curate their data, as well as preserve data after project completion. All data in non-digital formats will be stored in locked cabinets in secure facilities in Panama. Data will be managed in tiers: (1) data that will be made fully publicly accessible; (2) data that will be made publicly accessible in fully anonymised summary form; (3) data that will only be available to the immediate research team. At the end of the project, all non-digital data will be securely transported via an international courier service and securely stored at the University of Manchester for a minimum of five years after completion of the study. All digital data will be securely stored for five years in the University of Manchester RDMS. All data will be maintained in accordance with the Data Protection Act (1998).

### Data monitoring and quality assurance

A five-committee oversight structure will be adopted for the duration of the project. A Trial Management Group (including PI and CO-Is) will monitor all aspects of the conduct and progress of the trial and ensure protocol adherence. An Independent Project Steering Committee (IPSC) will provide oversight of the project throughout its various stages. An Independent Data and Ethics Monitoring Committee (DMEC) will review safety, quality and compliance. A Facilitator Engagement Group (FEG) will provide feedback on the trial and the process of delivering the programme, recruiting and assessing families. A Participant Engagement Group (PEG) will provide insight into what it is like to take part in the trial and will offer feedback to improve delivery, recruitment and assessment processes.

## Discussion

Systematic reviews suggest there is a gap in research on the effectiveness of family interventions in LMICs [40, 41]. The present trial will be one of the few rigorous evaluations of a family skills training programme in a LMIC, and the first, to our knowledge, to be conducted in the Central American region where interpersonal violence rates are high. The study intends to evaluate a well-known family programme that has been widely disseminated around the world.

The project includes a process evaluation that will allow exploration of factors that increase potential for sustained implementation. A fidelity analysis will explore whether the intervention was delivered as intended. Interviews with facilitators, trainers and site directors will examine the necessary conditions to ensure successful implementation and factors that increase families’ participation and retention. SFP 10–14 is a seven-session intervention and thus it is important to assess factors affecting implementation and receipt of its constituent components based on the intervention theory of change.

The cost-effectiveness analysis will be one of the few conducted in a LMIC. Understanding whether an intervention is good value for money is particularly important in low-resource settings. Together with process evaluation data, the cost-effectiveness analysis will answer whether the intervention is financially sustainable in the long term in this particular setting.

Data from this study have the potential to impact public policies for the prevention of interpersonal violence in Panama and the Region and provide valuable information for prevention strategies for LMICs. Our dissemination strategy includes sharing findings with local partners and international agencies.

### Trial status

At the time of submission of this manuscript, a total of 285 families have been recruited into the trial and assessed at baseline. Recruitment of waves 2 (*n* = 280) and 3 (*n* = 280) are expected to start in March 2018.

## Additional file

Additional file 1: SPIRIT 2013 Checklist: Recommended items to address in a clinical trial protocol and related documents*. (DOC 121 kb)

## Abbreviations

LMIC: Low and middle-income countries
MEDUCA: Ministry of Education, Panama
MINSA: Ministry of Health, Panama
SFP 10–14: Strengthening Families Programme 10–14
